# From the jaws of the “Leviathan”: A sperm whale tooth from the Valencina Copper Age Megasite

**DOI:** 10.1371/journal.pone.0323773

**Published:** 2025-05-14

**Authors:** Samuel Ramírez-Cruzado Aguilar-Galindo, Miriam Luciañez-Triviño, Fernando Muñiz Guinea, Luis Miguel Cáceres Puro, Antonio Toscano Grande, Marta Díaz-Guardamino, Juan Manuel Vargas Jiménez, Thomas Xavier Schuhmacher, Rafael María Martínez Sánchez, Santiago Guillamón Dávila, Joaquín Rodríguez Vidal, Leonardo García Sanjuán

**Affiliations:** 1 Department of Prehistory and Archaeology, University of Seville, Seville, Spain; 2 Canary Islands Oceanographic Center (COC), Spanish Institute of Oceanography (IEO), Spanish Research Council (CSIC), Santa Cruz de Tenerife, Spain; 3 Department of Crystallography, Mineralogy and Agricultural Chemistry, Faculty of Chemistry and Geology Museum, University of Seville, Seville, Spain; 4 Department of Earth Sciences, University of Huelva, Huelva, Spain; 5 Department of Archaeology, Durham University, Durham, United Kingdom; 6 Servicio de Arqueología, Ayuntamiento de Valencina de la Concepción, Valencina de la Concepción, Seville, Spain; 7 German Archaeological Institute, Madrid, Spain; 8 Department of History, University of Córdoba, Córdoba, Spain; Universita di Bologna, ITALY

## Abstract

During the excavations undertaken in 2018 at the Nueva Biblioteca sector of the Valencina Copper Age mega-site, in south-west Spain, an exceptional sperm-whale tooth was found inside a non-burial pit. This remarkable object is the first of its kind ever found for Late Prehistoric Iberia. Due to its rarity and importance, a multidisciplinary study was carried out, including photogrammetric 3D modelling, as well as taphonomic, paleontological, technological and contextual analysis. This led to a full characterisation of the artefact through the analysis of its bioerosion traces, anthropogenic marks, depositional context and socio-cultural background. The ensuing discussion covers the history and processes the tooth went through from the death of the animal and disposal on the seabed, through the disarticulation of the tooth to its collection in a coastal environment and its subsequent use and deposition in the pit.

'No permits were required for the described study, which complied with all relevant regulations.'

Not far from the gate is a common tomb, where lie all those who met their death when fighting against Alexander and the Macedonians. Hard by they show a place where, it is said, Cadmus (he may believe the story who likes) sowed the teeth of the dragon, which he slew at the fountain, from which teeth men came up out of the earth.

Pausanias, Description of Greece (Book 9.1-22), 2nd century AD

## Introduction

As a raw material, ivory has a long history of use that goes back to, at least, the Upper Palaeolithic [1–5]. Since Prehistory, it has been used to craft a wide range of (largely) sumptuous and sacred objects, ranging from personal ornaments (bracelets, necklaces, combs) to art (sculptures, figurines, furniture) or musical instruments [6]. Because of its physical properties, which render it fairly resistant to diagenetic processes, as well as aesthetic appeal and exoticism, ivory is a unique indicator in the study of ancient crafts, arts, exchange and socio-cultural organisation.

The large majority of the archaeological literature focuses on ivory from land mammals, mainly on proboscideans, but also on hippopotamus, deer, bear, wolf, etc. Thus, for the Iberian Peninsula, a vast bibliographic production reflects the presence of elephant ivory in Neolithic, Copper Age, Bronze Age and Iron Age contexts – for recent syntheses [6]. In recent years, this field of research has been expanded to include ivory (teeth) from marine mammals, particularly Odontocete cetaceans, but also Pinnipeds (seals, walruses, etc) and Sirenia. [7–13]. Undoubtedly, the use of such ivory may have been connected with the exploitation of marine mammals, a subject for which the available archaeological evidence is scant and difficult to interpret. In Africa, the evidence of the scavenging of whale carcasses goes back to the Lower Pleistocene [14]. In Europe, the processing and/or consumption of marine mammals, including cetaceans, is attested since the Upper Palaeolithic (Table 1) [15,16] and through the Mesolithic [17–20], Neolithic [12,21,22], Copper Age [10,11,23] and, of course, later historical periods [24–26]. Whether or not these animals were hunted in prehistory, or stranded carcasses were simply opportunistically exploited, has been and still is a matter of debate [27,28].

**Table 1:** Record of remarkable cetacean remains at archaeological sites in Europe belonging to Neolithic and Chalcolithic.

Material	Processing/ Consumption	Chronology	Location	Publication
Bowl made from a whale vertebra	Processed	3100-2200 bc, Neolithic	Skara Brae, Scotland	National Museum Scotland Collection
Whale bone carved figure	Processed	3100-2200 bc, Neolithic	Skara Brae, Scotland	National Museum Scotland Collection
Sperm whale tooth	Unprocessed	End of Neolithic, start of Copper Age	Sardinia, Italy	Melis y Zedda (2021)
Cetacean rib	Unprocessed	Copper Age	Leceia, Portugal	Cardoso (1995)
Cetacean rib	Unprocessed	Copper Age	Alpena, Portugal	Zbyszewski (1977)
Five buttons with V-hole perforation made from sperm whale teeth	Processed	Copper Age	Verdelha dos Ruivos, Portugal	Schuhmacher et al. (2009)
17 objects (beads, buttons and a cylinder) made from sperm whale bone	Processed	Copper Age	Praia das Maçãs, Verdelha dos Ruivos Palmela, Dolmen das Conchadas and Pedra de Ouro (Lisboa)	Schuhmacher et al. (2013)
Buttons with V-hole perforation made from sperm whale bone	Processed	Copper Age	Galera da Cisterna, Portugal	Zilhao (2016)
One double perforated button with appendages made of sperm whale teeth	Processed	Copper Age	Madrid	Liesau (2020)
Two vertebrae and a whale rib	Processed	Copper Age	La Vital, Gandía, Spain	Pascual Benito et al. (2019)

In this paper we present an exceptional sperm-whale tooth found at the Copper Age mega-site of Valencina de la Concepción-Castilleja de Guzmán (henceforth Valencina), located near the city of Sevilla, in south-west Spain. Following the discovery of La Pastora, a tholos-type megalithic monument, in the 1860s, and gradual, if slow, advances throughout the 20^th^ century, research on Valencina has made substantial progress in the last two decades. In addition to its large size (c. 450 hectares), Valencina is remarkable for the scale and number of features found in it, including massive ditches up to 9–10 m across and 8–9 m deep, remarkable *tholoi* (such as Montelirio, Matarrubilla, Structure 10.042-10.049 and La Pastora itself) as well as tens of thousands of pits, shallow basins and shafts used for a variety of purposes [for a recent overview, see [29]. Recent research has also highlighted the amount and quality of the material culture found in some of those features, particularly (but not only) in the *tholoi*. Of special relevance are the objects made in elephant ivory, which not only represents the largest collection of this raw material in 3^rd^ millennium western Europe, but also includes finely crafted and highly idiosyncratic artefacts [6,30–33]. Recent reviews of Neolithic and Copper Age Europe have discussed Valencina as a focus of early social complexity in 3^rd^ millennium Europe - see for example [34–36].

The sperm whale tooth studied here, found in excavations undertaken in 2018 at the Nueva Biblioteca sector of Valencina, is the first of its kind ever found in the Iberian Peninsula, and only the second published for the Western Mediterranean, after the one recently discovered at the site of Monte d’Accoddi, Sardinia [22]. A multidisciplinary approach is followed, including techniques and expertise from biology, geology, archaeology and taphonomy. This leads to a full characterisation of the item through the analysis of its bioerosion traces, anthropogenic marks and depositional context, followed by a discussion that places this find within the general background of the use of marine resources and ivory in Copper Age Iberia.

## Archaeological context

**Fig 1:**
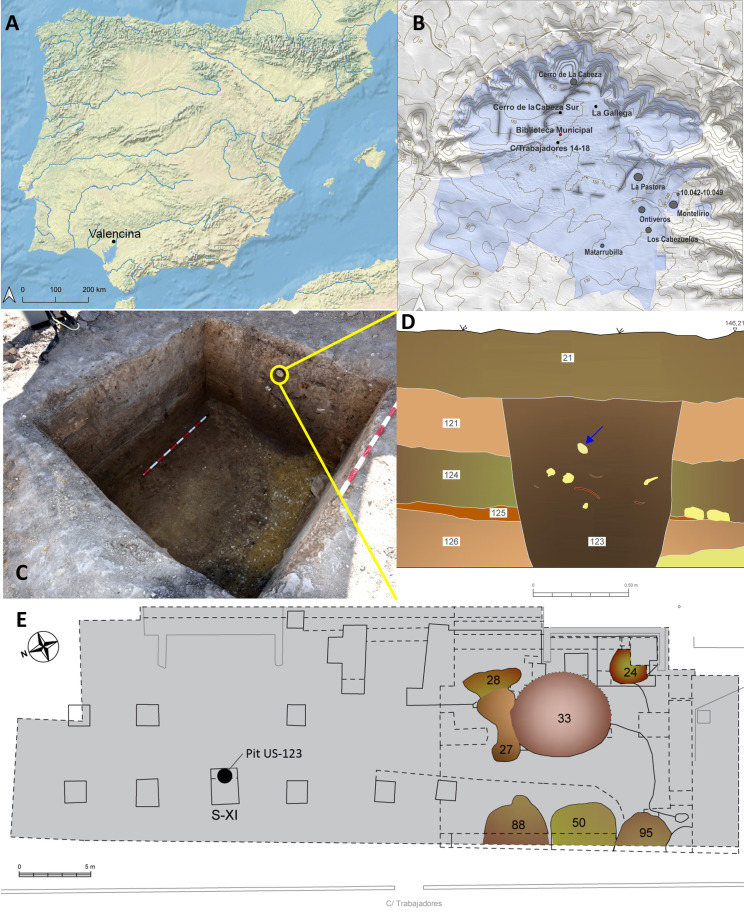
The Nueva Biblioteca sector in which the sperm whale tooth was found. A-B, geographical context; C, Pit US-123; D, stratigraphy of Pit US-123; E, general plan of the Nueva Biblioteca with the prehistoric structures discovered in this area (24,27,28,33,50,88,95: stratigraphic units).

The Nueva Biblioteca (‘New Library’) sector is located on the northern half of Valencina (Fig 1A–1C). Excavations undertaken during the spring and summer of 2018 as a result of the construction of a new municipal library revealed a series of features, including the pit in which the sperm-whale tooth described in this paper was found. Topographically, the Nueva Biblioteca land plot is at the foot of the western slope of a gentle hill. This hill, the highest elevation of the Valencina mega-site (158 masl), and designated as La Perrera, was excavated in the 1970s and yielded very interesting results, as is discussed below.

The Nueva Biblioteca excavation extended over an area of 758 m^2^ and revealed several interesting pieces of evidence. Prominent among those is the fact that stratigraphic deposits reaching an average depth between 1.20 and 1.5 m were found across most of the sector. This is quite remarkable, because a well-known characteristic of the Valencina mega-site is that the vast majority of the stratification occurs inside negative features (such as pits, shallow basins, shafts or ditches) that were cut into the local substrate of yellow sandy silts, while very little, or none, occurs outside those. The excavators were able to tell apart up to seven stratigraphic units, with three major phases of use and construction. In the earliest of those three phases, stones were used to build a feature circular in plan and measuring 28.79 m in length by 2 m in width, which was cut at three points by as many transversal ditches. This phase also included a single secondary inhumation inside a pit, measuring 1 m in maximum diameter, and involving a skull, a long bone and several other fragments (currently under study). The second construction phase involved the abandonment of the stone structure and the cutting of four shallow basins averaging 4 m of maximum diameter. The third phase involved the accumulation of clayish deposits over the entire area and new episodes of use, including five more pits. Two radiocarbon dates obtained for this study (Table 2) suggest a long period of use (c. 400 years) for the Nueva Biblioteca sector, which is in accordance with the deposition of thick layers outside underground structures as well as the diversity of features found across the sector, within a relatively small space, including three ditches, various stone features, pits, etc. The most recent of the two dates (MAMS 41417) place the third phase between c. 2500 and 2400 BC, that is to say, in the period leading to the abandonments of the Valencina mega-site (c. 2300 BC). It was at around this time that pit US-123 was made, and the sperm-whale tooth was deposited in it.

**Table 2:** Radiocarbon dates for the Nueva Biblioteca sector of Valencina.

LAB. ID	SAMPLE	CONTEXT	AGE BP	AGE CAL BC 2σ	ẟ^13^C AMS [‰]	C:N	C %
MAMS-41416	*Sus* (mandible)	UD 21 – Central strip	4049 ± 42	2848-2468	-37.6	3.5	4.2
MAMS-41417	Medium-sized land mammal (rib)	UD 21 – Central strip	3986 ± 24	2572-2463	-20.9	3.3	24.9

All calibrations with Oxcal 4.4 based on the IntCal20 Northern Hemisphere radiocarbon age calibration curve [37].

Pit US-123 was found on the eastern side of Trench XI and, initially, approximately only one-third of it could be excavated (Fig 1C and 1D). However, upon the discovery of the sperm-whale tooth, the trench was extended to allow a fuller excavation. At the end, although the pit could not be excavated completely, its shape and measurements were established with a reasonable degree of certainty. On its upper part it had a circular plan c. 1 m in diameter, narrowing down to the bottom to 64 cm, which resulted in a conical shape. Its bottom could not be reached, the maximum recorded depth being 89 cm. The sperm-whale tooth was found on the upper third of the pit’s infill, consisting of a dark brown clayish soil with some small sandstone. No evidence of combustion was found inside the pit.

The pit contained 187 pottery fragments, weighting 3.437 kg, which in general did not show a high degree of rounding. This suggests that the pottery fragments did not experience a long period of wearing before being introduced into the pit. The fragments whose shape could be discerned (34 in total) reveal the habitual pottery repertoire at Valencina, with large ‘almond-rim’ dishes (with diameters up to 40–50 cm), saucers as well as smaller globular vessels and bowls – see a recent review [38]. It is worth noting a fragment decorated with strokes of black paint on reddish coating, of a kind well known in Valencina [39], which also shows two perforations. A fragment of an ‘almond-rim’ plate also showed three perforations, plus a fourth, unfinished one, which were placed apparently randomly. The concentration of four perforations on a single pottery fragment is a very rare occurrence at Valencina and seems to suggest some kind of ‘drilling’ practice, rather than the need to repair the vessel. Indeed, the presence of two fragments with perforations in a single pit is also quite infrequent at the site. In general, it is intriguing that this ‘abundance’ of perforations on pottery should occur in a pit containing a sperm whale tooth that shows obvious signs of perforation (see discussion below).

Pit SU-123 also contained seven fragments of macro-lithic tools, possible grinding stones of the kind well recorded at the site [40] as well as one small, knapped blade, probably in quartzite. The dearth of knapped lithics in this pit is also quite remarkable, considering how frequent these items are across the site.

Finally, Pit SU-123 also contained 87 animal skeletal remains. They are in a very good state of preservation, showing excellent chemical integrity, as is usual in the bone remains found in the Nueva Biblioteca sector. The zooarchaeological analysis revealed a total of 26 taxonomically identifiable remains (Table 3).

**Table 3:** Number of identified remains per taxon (NISP) and unidentified remains per group (NR) and weight of remains (WR).

UD 123	*Bos taurus*	*Capra hircus*	*Ovis aries*	Caprinae	*Sus scrofa*	LSM	MSM	TOTAL
**NISP/NR**	4	9	1	1	11	19	42	87
**WR (g)**	485,99	52,1	7,16	3,75	92,03	102,4	95,35	838,78

**LSM,** Macromammals; **MSM,** Mesomammals.

With regard to the taxa identified, these were composed entirely of domestic mammals related to livestock activity, including domestic cattle (*Bos taurus*), goats (*Capra hircus*), sheep (*Ovis aries*), indeterminate goats (Caprinae) and swine (*Sus scrofa*). The latter taxon predominates in the total determined assemblage, as appears to be the case in the entire Nueva Biblioteca zooarchaeological assemblage. No marine mammals were identified. All of the remains were completely devoid of cutting or splitting marks linked to quartering. However, in at least three of them, fractures made in fresh bone were observed. More than 50% of the identified remains of land mammals (23, Table 3) showed signs of thermo-alteration, ranging from relatively low temperatures, with yellowish or brownish colourations, to relatively high temperatures with completely calcinated bones. The latter is mainly observed among the pig remains, and also exists in one of the undetermined caprine remains.

The soil forming the infill of pit US-123 was very homogeneous, and no strata, nor specific features, were observed in it. Therefore, this infill is interpreted as anthropogenic in nature and resulting from a single event, in which a series of items (pottery, lithic tools, animal remains and the sperm-whale tooth) were deliberately buried as a structured deposition (or offering) as a result of a single event, probably ceremonial in character.

## Material

**Fig 2:**
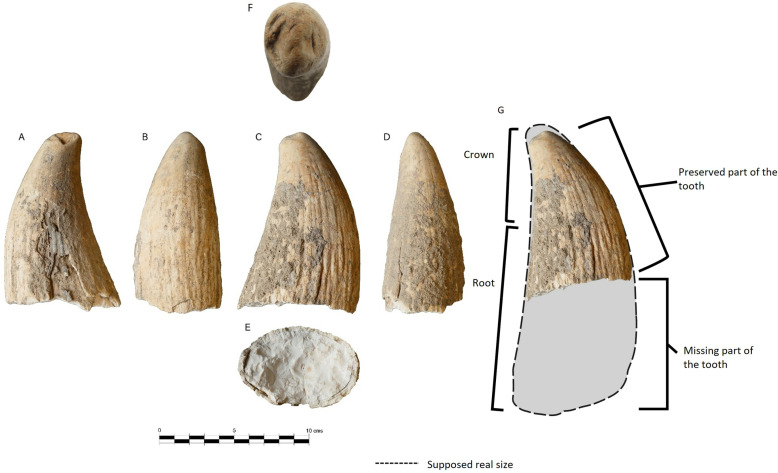
The tooth of sperm whale studied. A: lingual view, B: mesial view, C: labial view, D: distal view, E: root section detail, F: occlusal view; G: reconstruction with a complete tooth. Shaded area = missing part of the tooth. A high resolution 3D model of the tooth can be viewed and downloaded here: https://skfb.ly/ovWY6.

The specimen studied here is currently kept in the Valencina Municipal Museum and tagged as [Nv. Biblio. 2018/13-S. XI-UD.123-nº 1]. It corresponds to an incomplete isolated sperm whale tooth (preserving approximately its upper half) in a good state of preservation (Fig 2).

## Methods

Since the tooth is the first osseous remain of a marine mammal ever found at Valencina, and because of its special character, it has been studied using various methods in order to obtain as much information as possible about its origin, transformation and cultural significance.

A 3D model of the tooth was made in line with recent advances in the field of 3D imaging and digital technologies for the study of archaeological artefact, which have led to the development of digital archaeology, favouring non-invasive studies, avoiding excessive manipulation and providing information enabling the visualization and examination of very subtle details that may be invisible to the naked eye [41–44]. Photographs were taken with a Nikon d3400 camera equipped with a Nikkor af-p 18–55 mm lens. The photogrammetric processing was carried out using Agisoft Metashape software available at the computer services of Durham University. Post-processing phase involved the analysis, treatment, fit to the plane, orientation and scale assignment to the textured photogrammetric models. The image analysis was carried out using Agisoft PhotoScan. Applying various algorithms and false colour analysis in MeshLab and CloudCompare, it was possible to emphasise the morphology of the intended tooth surface, facilitating its visualisation, analysis and interpretation (the resulting 3D model can be viewed and downloaded here: https://skfb.ly/ovWY6). This work made it easy to interpret and visualize the tooth without having to handle it, thus minimizing the risk of damaging it, as well as to be able to present the tooth to the media or exhibit the 3D replica in the museum instead of the real one.

To identify possible traces of anthropic manipulation, the tooth was examined with a ShuttlePix P-400R digital microscope (up to 400X) from the University of Seville´s CITIUS microanalysis service. The description of the technical surface traces and the general vocabulary for the technology were based on Averbouh and Provenzano [45] and the Multilingual Lexicon of Bone Industries [46].

Anatomical tooth terminology based on Bianucci & Landini [47], Toscano [48] and Lambert & Bianucci [49].

Bioerosion traces were observed by using the digital microscope ShuttlePix P-400R (up to 400X) and taking photographs with the PowerShot SX50 HS camera, which made it possible to identify different ichnogenus caused by the action of marine organisms, which respond to different behaviours. Moulds were also made of some of the traces. The whole process was carried out at the Valencina de la Concepcion Museum.

## Results

### Description

The fusiform morphology of the tooth as well as its large size and massive and robust root allow it to be attributed to the superfamily Physeteroidea. The absence of a rough enamel crown, constriction or occlusion facets or wear by opposing teeth places it within the family Physeteridae and subfamily Physeterinae [50,51] differentiating it from the so-called macroraptorial sperm whales. The tooth was compared with specimens described in the literature and with material from various collections and online 3D models, showing great similarity with teeth belonging to present-day sperm whale *Physeter macrocephalus.*

The tooth is robust, unicuspid, conical, gently curved lingually and distally, with an oval cross-section, more compressed labio-lingually. It would have had a symphyseal mandibular position, probably right (the labial side is usually more curved or convex than the lingual side, resulting in a gentle inward curvature of the mouth). Approximately the upper half of the tooth is preserved, measuring 13.2 cm with a weight of 414 g. Lambert [51] indicate that the largest *Physeter* tooth measured was 25 cm. This suggests a tooth that would be close to that size in its entirety. In addition, the tooth shows features like wear on the labial side, and a smoothed fracture with loss of material on the lingual side (see Fig 2A–2C) that indicate that it was produced during the animal’s life, either during feeding or by collision with the teeth of a rival (clashes between males), so it belonged to an adult specimen that may have died naturally.

The crown is smoother on the surface than the root. The enamel appears to be absent, showing a thin, polished cementum layer that allows the dentine to show towards the apex. The tip shows wear on the labial side, while on the lingual side there is a fracture with loss of material forming a slight hollow with rounded edges. Its passage to the root is not perceptible. Neither is the banding of the gingival or alveolar margin or any constriction visible.

The root of the *P. macrocephalus* tooth is massive, fusiform, and widens until it reaches a more dilated shape in the middle part, before flattening and compressing in its lower half, which projects distally. In the tooth under study, the lower fracture of the preserved part shows a stable to slightly increasing diameter which places us in the middle part of the tooth. The size of the preserved part and its total estimate would indicate a large adult specimen. The root has a thick layer of cementum, with an outer ornamentation of folds and parallel grooves in a baso-apical orientation that deepen towards the base, and which are related to the anchorage of the tooth to the alveolus. This striation crosses transversely with a certain undulation corresponding to growth layers. The lower part, which is absent in the specimen studied here, has been separated by a net and level fracture in a cross section that shows no signs of environmental wear, preserving well-defined angles. This basal fracture allows the different tooth layers to be observed in section. The tooth grows as a superposition of cones that are added towards the base. In cross-section, this structure of overlapping layers looks as concentric rings that are clearly visible. Although the conical opening of the pulp cavity is not preserved, as the base of the root is not present, a small pulp canal or root canal through which the vessels and nerves of the tooth run is still preserved in its centre. Surrounding it are concentric layers or rings of dentine, which make up the bulk of the tooth material (maximum dentine thickness: 30 mm). Towards the outside, a layer of cement appears (maximum cement thickness: 6 mm), with a mineralisation that is more granular in appearance than the dentine, and which in the specimen is shown subdivided into 2, where the outermost and thinnest layer is detaching from the tooth. These detachment or fragmentation layers usually form at the separation between the different cementum accretion lamellae.

The dentine is compact and white in colour, almost like a fresh tooth. It shows alteration on the exterior surface, where traces of sediments and concretions can be found. Alterations produced by microorganisms, as well as loss of raw material and some generalised cracks in the cementum are also visible. However, the tooth is not complete, and an important part of the proximal end is missing. Its maximum diameter (approx. 8 cm) would point to a medium-sized tooth, about 20 cm long. The estimated size, together with the lack of pulp cavity in the preserved part, lead to think that about 60% of the tooth is preserved (Fig 2G)

The morphological study of the tooth has allowed us to identify it as belonging to a Sperm whale (Physeteroidea), a superfamily of odontocete (toothed) cetaceans known since the Upper Oligocene (around 25 million years BP) [52–54]. While they diversified enormously during the Middle and Upper Miocene [47,48], they are currently represented by only three species, belonging to two families: the Physeteridae family, with *Physeter macrocephalus* Linnaeus, 1758, being this the only member of the genus and whose adult males can reach up to 20.5 meters and weigh up to 57 tonnes; and the Kogiidae family, with two smaller species *Kogia breviceps* Blainville, 1838 (some individuals can reach up to 3,5 m and 400 kg of weight) and *Kogia sima* Owen, 1866 (the smallest species with a length of 2,7 m and 280 kg of weight)*.*

Sperm whales are oceanic animals, frequenting both the depths of the continental slope and coastal waters. They have a global distribution and are frequent in the Atlantic and Mediterranean waters of the Iberian Peninsula. Today, they come to the Strait of Gibraltar to feed on squid, mostly in spring and early summer, and are considered a semi-resident species [55,56].

Although present-day sperm whales, *Physeter macrocephalus*, have a cosmopolitan distribution, there are few mentions of fossil remains from after the Neogene. According to the Paleobiology Database portal, there are Holocene archaeological records from Spain, Portugal, Morocco and Italy [10,21,57].

### Invertebrate bioerosion traces

**Fig 3:**
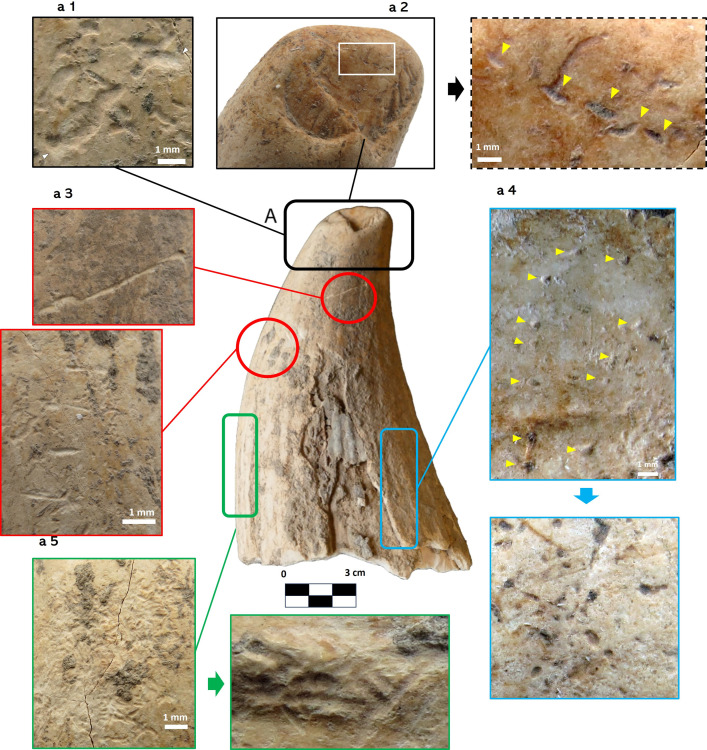
A; location of invertebrate (rectangles) and vertebrate (circles) ichnogenus. Lingual view of the tooth. Bioerosion is not delimited to the marked areas, they represent examples of some of its locations, black a1 *Meandropolydora*, black a2 *Rogerella*, red a3 *Linichnus*, blue a4 *Entobia*, green a5 *Radulichnus*.

Four types of bioerosion traces were identified that are related to the boring activity of different groups of marine invertebrate organisms, corresponding to the ichnogenus: *Entobia* Bronn, 1838; *Maeandropolydora* Voigt, 1965; *Radulichnus* Voigt, 1977 and *Rogerella* De Saint-Seine, 1951 (Fig 3). For each ichnogenus, the main parameters of distribution in the tooth and the relative bioerosion rate are given, the values of the latter being very abundant >70%, abundant 70–40%, poor 40–10%, very poor <10% (Table 4).

**Table 4:** Main parameters of distribution in the tooth and the relative bioerosion rate.

Ichnogenus	Relative bioerosion rate	Ethological category	Trace maker
*Entobia*	poor 40–10%	Domichnia	Clionid sponge
*Maeandropolydora*	very poor <10%	Domichnia	Polychaete annelid
*Radulichnus*	poor 40–10%	Pascichnia	Gastropod/polyplacophoran
*Rogerella*	very poor <10%	Domichnia	Acrothoracican cirripedes

### Entobia

**Diagnosis:** This ichnogenus is defined as boreholes composed of a single chamber or several chambers linked by a network of galleries connected to the surface by numerous openings [58,59].

**Description:** In the Valencina tooth, they have been diagnosed thanks to the presence of numerous perforations corresponding to chamber openings, no more than 1 mm in diameter (Fig 3: a4), on its surface. Their relative bioerosion rate is poor 40–10% (Table 4).

**Producer and environmental data:** These structures are related to the activity of members of the sponge family Clionaidae and are ethologically interpreted as a domichnion (dwelling and/or dwelling structure) [58,60]. This bioerosion structure is also an indicator of clean waters, without suspended sediments, typical of bays and rocky cliffs [59].

### Maeandropolydora

**Diagnosis:** These structures correspond to long cylindrical galleries, with two or more openings, which run along the substrate in sinuous or irregular contortions [60,61].

**Description:** On the surface of the tooth there are grooves (due to the disappearance of part of the affected enamel), no more than 1 mm wide and several mm long, which run along the surface of the tooth in a sinuous path (Fig 3: a1). They are concentrated in the distal area of the tooth, being their relative bioerosion rate very poor <10% (Table 4).

**Producer and environmental data:** From an ethological point of view, it is interpreted as a dwelling structure (domichnia) possibly carried out by the drilling activity of polychaete spionid annelids [60,61].

### Radulichnus

**Diagnosis:** According to Gibert [62] it consists of a set of shallow grooves of millimetre dimensions, short, parallel or subparallel and arranged in rows or criss-crossing. Each set of grooves reflects a “grazing trace” of the producer as it scrapes the surface with the radula to feed on algae attached to the surface of either bone or shell and each groove corresponds to a denticle of the radula [62,63]. The different patterns in the grooves reflect different types of radula and different producing organisms.

**Description:** In our case, they are observed clustered in different parts of the tooth surface, mostly in the central area, forming a mesh of grooves of 1mm long (Fig 3:a5). Their relative bioerosion rate is poor 40–10% (Table 4).

**Producer and environmental data:** This ichnogenus is related to the grazing activity of herbivorous gastropods or polyplacophores, ethologically interpreted as pascichnia [62].

### Rogerella

**Diagnosis:** This ichnogenus consists of elliptical, comma-shaped boreholes with a narrower distal portion, sometimes with a slight curvature and a circular proximal portion [64,65].

**Description:** In our case, they are observed as longitudinal sections in the shape of a pouch, with an elliptical outline and a narrower distal portion. They are short, 1 mm long (Fig 3:a2). They are concentrated in the apical area of the tooth, being their relative bioerosion rate very poor <10% (Table 4).

**Producer and environmental data:** They are made by acrothoracic barnacles in search of a home, interpreted ethologically as a domichnion [66].

### Vertebrate bioerosion traces

Other bioerosion structures have been observed, produced in this case by vertebrates. Specifically, bite traces, possibly produced by fish (chondrichthyes or osteichthyes) were found. In ichnology, structures resulting from trophic interactions between fish (e.g., sharks) and marine mammals (mainly whales) are known as ichnogenus *Linichnus* [67].

**Fig 4:**
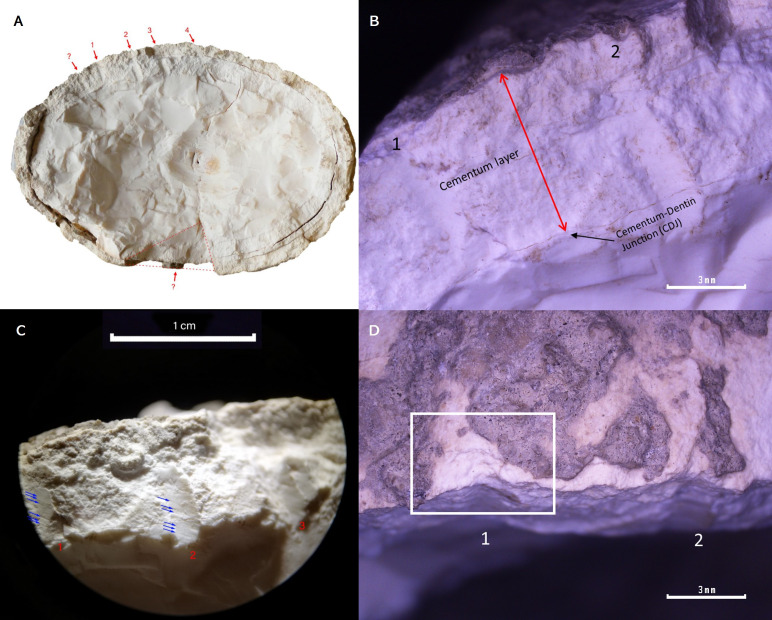
A; View of the transverse fracture. Indication of the traces and striations mentioned in the text. **B;** Localised extension of the traces in the cementum layer. Only groove Nr 2 has several millimetres of travel in the dentine. **C;** Location and trajectory of the observed grooves. **D;** Possible flake-like detachment.

The bite traces observed on the Valencina sperm whale tooth correspond to the ichnospecies *Linichnus bromleyi* [68]. This ichnospecies corresponds to a single groove, straight or slightly curved, with a non-serrated edge, a lanceolate morphology and V-shaped in cross section [68]. They are interpreted ethologically as the result of a sharks´s biting behaviour on a marine mammal (Fig 4: a3).

The bite traces appear in two areas, and both correspond to the teeth of each jaw of the producer. The dimensions of the longer trace are 1.58 cm long. They were possibly produced by sharks during the scavenging action of the carcass of the sperm whale. It is not possible to identify which bite trace corresponds to which jaw of the shark.

### Anthropic marks

Observation through the microscope led to the identification of marks and traces that are not comparable with those made by biological action. Several traces have been observed in the transverse fracture and have not been identified elsewhere on the tooth.

Four straight grooves, sub-parallel to each other, can be seen on one side of the fracture (Fig 4A). A fifth possible, but unclear, trace is observed next to trace #1, together with a set of striations in the opposite area. The latter set of grooves could be associated with a large triangular-shaped detachment of material. The grooves are clearly visible in the cementum layer, but their trajectory or extension is not clearly visible in the dentine. A certain path of the groove beyond the cementum can only be seen in impression No. 2 (Fig 4B).

## Discussion

The multidisciplinary approach used for this study has led to new evidence for interpreting the remarkable sperm whale tooth discovered in Valencina.

**Fig 5:**
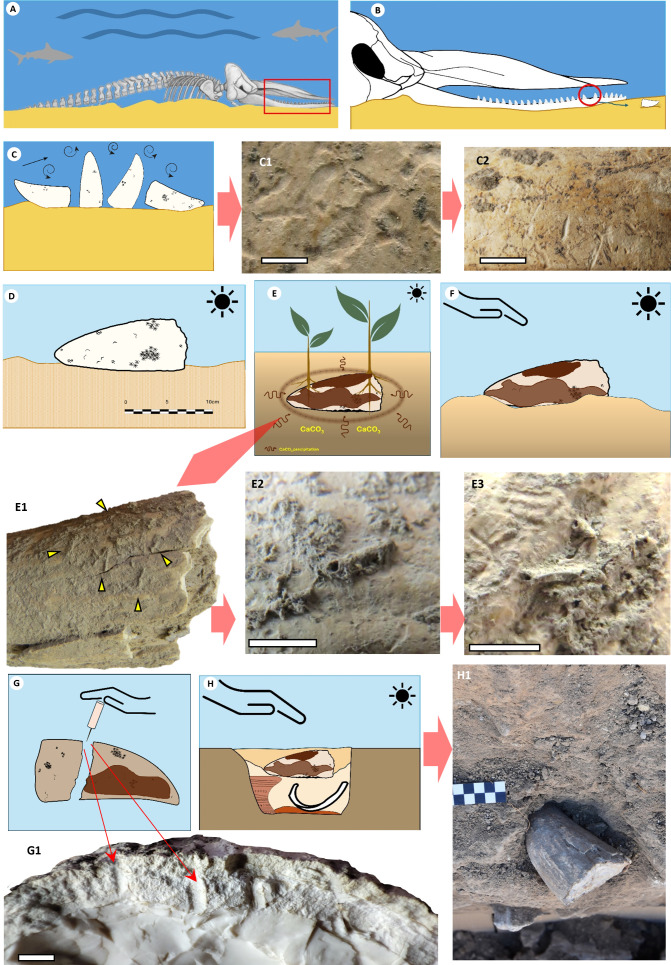
Story telling board representing the processes the tooth has undergone from the death of the sperm whale and its deposition on the seafloor (A), to its discovery in Pit US-123 of the Nueva Biblioteca in 2018 (H). **A:** hypothetical situation of the sperm whale skeleton on the seafloor; **B:** detail of the jaw and disarticulation of the tooth; **C:** stage of transport and colonisation of the tooth; **C1:** *Meandropolydora*, **C2:** *Linichnus*; **D:** deposition of the tooth in a beach environment; **E:** shallow burial and start of the continental bioerosion process by plant roots and creation of the crust covering the tooth by precipitation of CaC03, **E1-E3:** structures left by the roots covered by the crust, it can be seen that these are on top of the previous marine bioerosion traces, arrow: root; **F:** exposure of the tooth and collection; **G:** manufacture and extraction of parts of the tooth for its possible use, **G1:** detail image of the marks left by the tool used for its manipulation; **H:** deposition of the tooth in the pit US-123, **H1:** the tooth as was discovered inside the pit. Scale C1, C2, E2, E3 and G1: 5mm.

The bioerosion traces (Fig 5) suggest that the tooth surface was colonized by marine organisms such as sponges and gastropods. This scenario would have been possible only if the tooth had remained for some time on the seafloor, either still attached to the whale’s jaw during the early phases of carcass decomposition or isolated after separation of the mandibular alveolus (Fig 5A). Either way, the tooth served as a hard substrate where different types of organisms bioeroded. The bite traces, probably from sharks, originated during the scavenging that followed the animal´s death. There are bioerosion traces on all sides of the tooth, which would indicate that, after it separated from the jaw, the tooth was transported along the seafloor by ocean currents, with periods of exposure without rolling (bioerosive phases of invertebrates), subsequently being totally or partially buried (Fig 5C).

After this time under water conditions, the tooth washed ashore, where it was exposed to subaerial conditions (Fig 5D). It is very likely that it arrived to the shore as part of an energetic event (storms or large tides), which usually bring to the shore elements more typical of deep areas. This could be the case also of the *Pecten maximus* (scallop) shells that have been found in other parts of Valencina. Pecten shells are typical of deep environments up to 150 m [69] and appear on the coasts after energetic events, as could have been the case with the tooth. During this time, the tooth was covered by sediment again, and it underwent another weathering process, in this case affected by the action of roots and the creation of a cemented crust (Fig 5E). After this process the tooth reached the surface, when it was collected by people (Fig 5F). After manipulation, perhaps to extract raw material (Fig 5G), and an undetermined period of use, the tooth was deposited in a pit, where it remained buried for over four thousand years (Fig 5H).

The evidence described above suggests that this dental piece did not result from hunting or from the harvesting of the carcasses of stranded whales. In neither of these cases, would the tooth present the bioerosion traces made by colonising organisms described above. In fact, although there is evidence of the use of whale parts at various Portuguese sites [10,23,70,71] there is no record of active whaling by Chalcolithic societies in southern Spain. Cetacean hunting by Mesolithic and Neolithic societies is documented in Northern Europe [18,72], and South Korea [73] among other regions. The absence of further bone remains belonging to a sperm whale (or other marine mammals) at Nueva Biblioteca also runs counter to the possibility that this animal was hunted by the inhabitants of Valencina. A large trophy like a sperm whale is usually followed by remains of bones and objects made with them, such as harpoons and tools [18].

In relation with the anthropic marks the imprints are flat-walled with straight and oblique grooves (Fig 4C), which, contrary to what would be expected if the grooves had been the product of a rotational drilling procedure, would leave cylindrical or semi-conical grooves (depending on the tool) with concentric or spiral grooves on their surfaces. A scale-shaped detachment of raw material has been observed on the fracture edge associated with groove No. 1 (Fig 4D). However, it is very small, which would indicate that it was either a soft percussion or rather minimal continuous pressure to insert a tool. The orientation of the striations, as well as the group of striations and the triangular detachment on the other side, would point to the same hypothesis (sustained pressure). On the one hand, the four flat-walled grooves indicate the insertion of several tools of the same type, in order to weaken the area and generate a fracture line. Due to the shape of the grooves, the use of a round-section awl is ruled out, pointing rather to a square-section awl or a narrow chisel. The orientation of the striations (see Fig 4) could indicate an unintentional sliding of the tool inwards and sideways at the moment of the separation of both tooth segments. On the other hand, the large dentine detachment on the opposite side and the associated striations could indicate the insertion of a larger (wider and thicker) wedge-shaped tool, which would print an opposing force.

The observed marks are difficult to interpret because they were only preserved in the cross section of the tooth, since the other traces would be found in the missing fragment. Precisely, the observed segmentation procedure is not clear due to the partial nature of the preserved stigmata. It was not possible to determine the raw material of the tool used, not only because of the lack of a part of the tooth but also because the stigmata were erased by post-depositional processes. Nevertheless, it can be said that the separation of the two tooth fragments would not have involved a violent blow, but rather a contained pressure through the insertion of several tools exerting force in a convergent manner until the material was separated.

According to the technical expertise observed among Iberian 3rd millennium BC societies, it is more likely that the marks observed have to do with a separation process to use the tooth as an artefact (e.g., segmentation for better handling, manufacture of supports, etc.), rather than traces of an initial extraction procedure.

In terms of the social and cultural significance of the tooth, the archaeological context in which it was found is as interesting as the tooth itself. Nueva Biblioteca is surrounded by several sectors of the Valencina site that have been excavated since the 1970s, including (clockwise) Valencina Nord, Cerro de la Cabeza, La Gallega, La Perrera and Calle Trabajadores. At the ‘Valencina Nord’ sector, located about 300 m to the North of Nueva Biblioteca, geophysics and excavations undertaken by the German Archaeological Institute of Madrid since 2014 have revealed several features, including large ditches and negative features (pits, basins) undercut in the bedrock, which in some cases have been interpreted as dwelling floors [74–76]. The Cerro de la Cabeza excavations made in the late 1970s yielded what, with 17 items, still amounts today to the largest collection of figurines and ‘idols’ ever found at Valencina, including two full-bodied anthropomorphic figurines made in bone which were found as part of the infill of shaft #1 [77]. Indeed, Cerro de la Cabeza accounts for half of the collection of portable imagery recorded at Valencina, which a recent review set in 35 items [78]. Further to the north-east, at a distance of c. 650 m from Nueva Biblioteca, excavations carried out in 1990 at La Gallega sector revealed 23 features cut into the marly bedrock (mostly pits, shallow basins and post holes), one of which yielded human remains [79]. The associated material culture included two elements of portable imagery: one engraved plaque (a rare find in Valencina) and small cylinder made in bone, both of them showing the ‘oculus’ motif that is pervasive in Late Neolithic and Copper Age Iberian imagery [79]. Later, a study of some of those pits suggested they were compost-making devices intended to support agricultural production [80]. At La Perrera, located c. 150 m to the north-east of Nueva Biblioteca, excavations undertaken in the summer of 1975 under the auspices of the Sevilla Archaeology Museum, revealed a large ditch, 8 m in depth. The infill of this ditch yielded what the excavators described as an ‘immense wealth’ of artefacts [81] including large amounts of pottery and faunal remains as well as various human inhumations among which it is worth noting an individual buried under a cairn of stones, without grave-goods [81,82].

Finally, barely 90 m to the south of Nueva Biblioteca it is worth noting the excavations carried out at Calle Trabajadores Nº 14–18 in 2008, which led to the discovery of 30 negative features, predominantly circular in plan, and of varying sizes and depths [83]. A total of 20 radiocarbon dates obtained for four of those features showed that activity at this sector started between 2580 and 2465 and ended between 2470 and 2310 cal BC 2σ, placing it at the end of the sequence of occupation of the site [84]. The relatively high frequency of Beaker pottery at Calle Trabajadores Nº 14–18 is consistent with that chronology. The features unearthed at Calle Trabajadores Nº 14–18 yielded some remarkable finds such as three large-sized copper axes (ranging between 20.5 and 34.9 cm in length) forming what the excavators described a ‘closed deposition’ in Structure UE-56 [83], 21 macrolithic items (including 14 quern fragments, 4 hammers/grinders, 2 hammers and 1 grinder), the largest assemblage of such tools so far recorded at Valencina, were found in association with a human skull and mandible in Structure UE 136 [40]. A recent study counted 1774 lithic items from this excavation, including knapped tools, debris and undetermined objects made in basalt, flint, rock crystal and green rhyolite [85].

Altogether, located on the northern half of the site, the Nueva Biblioteca sector is surrounded by a highly dense, complex and diverse arrangement of elements that includes hundreds of features and a vast amount of artefacts. As well as average-sized pits (about 1 m in diameter and depth), shallow basins (up to 4 m across), deep shafts and large-sized ditches (up to 8 m in depth), the area also includes a large megalithic monument (Cerro de la Cabeza). The functional interpretation of many of those features is not straightforward. Some of La Gallega pits have been interpreted as compost-making devices and storage facilities, while some of the shallow basins at Valencina Nord have been interpreted as ‘hut floors’ displaying post holes, light architecture in timber and sun-dried mud. However, some of the pits and shallow basins at La Gallega [79–80] and Calle Trabajadores Nº 14–18 [83] (and, notably, the La Perrera ditch) [81], were used for burial, while some others appear to have been used for structured deposition and were votive in character. The most remarkable example of the latter is Structure UE-56 at Calle Trabajadores with its three massive copper axes, which were not associated to human remains (that is to say, they were not intended as grave goods).

This leads us straight into the heart matter of the functional significance of the pit the sperm-whale tooth was found in. The absence of human remains strongly suggests the pit was the result of a structured deposition, sensu Chapman, 2000 [86], a widespread phenomenon in Neolithic Europe [87–89]. In other words, it was intended as a votive offering. This places the sperm-whale tooth in line with other highly-valuable and symbolically-charged artefacts found in non-funerary features of Valencina. An example of this is the gold foil decorated with ‘oculus’ motifs found in Structure 10.029 (a non-funerary pit) at the PP4-Montelirio sector (located c. 2.5 km to the south-west of Nueva Biblioteca) [90] which is the largest gold item ever found in Copper Age Iberia. Another example is, the three large copper axes of Structure UE-56 at Calle Trabajadores [83], very near the Nueva Biblioteca sector, described above. By being introduced in underground features and then buried, such special (in fact, unique) items were symbolically ‘destroyed’, and withdrawn from mundane life. Given their uniqueness, high intrinsic cost and, presumably, high social value, which made them inalienable this must have involved highly-staged performances. In other words, this tooth was treated as a totemic and/or sacred object. Plenty of examples exist in Valencina of how the material remains of large animals (elephant tusks, ostrich eggshells, deer antler, auroch horns, etc.) were treated with the utmost reverence, often being used as grave goods of socially prominent individuals. The worldview of Iberian Copper Age societies clearly included a high consideration of large, powerful animals. In addition, the examples mentioned above leave no doubt as to how extremely valuable objects (such as the decorated gold foil from Structure 10.029 of the PP4-Montelirio sector or the three large copper axes of Structure UE-56 at Calle Trabajadores) were sometimes buried in simple pits without human remains, as a form of structured deposition with a high symbolical character.

Two attempts to radiocarbon-date the sperm whale tooth failed on account of low collagen. The reasons behind the (apparently high) variability in the amount of collagen in human and animal bones of Copper Age chronology at the Valencina mega-site are basically unknown, although presumably they are connected to spatial variations in taphonomic and pedological conditions. A good example of the limitations imposed by this factor is Structure 10.049, the grave of ‘The Ivory Lady’ [91]. Five dates originally obtained from samples from the various ivory artefacts found in this tomb revealed very low collagen levels and yielded inconsistent ages that were deemed invalid [92]. A further seven samples of human bone and ivory submitted later, produced little or no collagen and failed the Oxford and SUERC (Scottish Universities Environmental Research Centre) quality-control procedures [84]. Finally, three more samples on human bone submitted to SUERC in 2022 also failed because of low collagen. In total, 15 samples from Structure 10.049 have been submitted for radiocarbon dating between 2012 and 2022, all of which failed due to low collagen. In this respect, four samples of approximately 1 g were extracted from the tooth core and sent to SUERC radiocarbon dating facility, and other two to BETA Analytic Laboratory. Unfortunately, all samples failed to provide sufficient quality collagen to proceed with an AMS measurement. Despite the lack of direct radiocarbon dating, the assemblage of materials found in connection with the tooth, including the fragments of almond-rim plates coupled with the most recent of the two dates (MAMS 41417) obtained as part of this study, leaves no doubt as to its usage in the final centuries of the Copper Age.

The fact that the tooth is not complete and shows fractures caused by anthropic action might suggest that some of it was used as raw material, in order to manufacture sumptuary objects, such as ornaments, etc. Although, no ivory artefact has yet been diagnosed as coming from a sperm whale tooth, recent finds of ivory of marine origin in European archaeological contexts have brought a new focus to the study of the use of marine resources by prehistoric societies [10–12,16].

Indeed, the discovery of another sperm whale tooth, very similar to the one described in this paper, in Monte d’Accoddi [22] may help to understand the use and symbolism of the Valencina tooth. Both were found with no association to other remains of sperm whale or other marine animals, and both were found in what are clearly special places for their respective contexts: a votive offering (Pit US-123) at Valencina and a massive ritual monument at Monte d’Accoddi. Additionally, both teeth show marks of anthropic manipulation, which would indicate an intention to extract raw material, perhaps to manufacture objects, or to dress them to make them more suited for their intended use.

In summary, it seems likely that this piece arrived to Valencina as an exotic product, as did other raw materials of great value (such as flint, ivory, rock crystal, cinnabar, ostrich eggshell and amber), and that it was collected on a marine shore rather than extracted from a hunted whale.

### Conclusions

The sperm whale tooth studied in this paper is the only of its kind ever found in Copper Age Iberia. In fact, the only example of a similar chronology and morphology in Europe was recently found in Sardinia.

This tooth, belonging to an adult sperm whale specimen, was likely found in a coastal area after it had spent some time in subaquatic conditions, as attested by the bioerosion traces left by marine organisms. Once the tooth was exposed on the surface, it was covered again by sediment and a second wearing process started, this time due to the action of roots and the creation of a cemented crust covering the tooth. After its collection, it was manipulated, perhaps with the aim of using some parts of it as a raw material to manufacture other objects (such as personal ornaments) or to transform it into a symbolically-charged artefact. Finally, the tooth was deposited in a pit.

The discovery of this piece underlines the presence of the sea in the worldview of the communities that lived or frequented Valencina in the 3^rd^ millennium BC. This is further suggested by the careful selection of rocks naturally ‘decorated’ with sedimentary structures of currents, bioerosion and marine organic remains, to manufacture some of the capstones and floor slabs used at major megalithic monuments such as La Pastora or Matarrubilla [93,94] in the use of numerous valves of *Pecten maximus* as offerings in several of the burials and votive pits that were made around ‘The Ivory Lady’ burial, in the PP4-Montelirio sector [95] or in the use of tens of thousands of discoidal beads to manufacture the attires worn by the individuals buried in the Montelirio *tholos* [96].
